# Physicians’ Use of the Computerized Physician Order Entry System for Medication Prescribing: Systematic Review

**DOI:** 10.2196/22923

**Published:** 2021-03-04

**Authors:** Asra Mogharbel, Dawn Dowding, John Ainsworth

**Affiliations:** 1 Division of Informatics Imaging and Data Sciences, School of Health Sciences, Faculty of Biology, Medicine and Health, Manchester Academic Health Science Centre Centre for Health Informatics The University of Manchester Manchester United Kingdom; 2 Division of Nursing, Midwifery and Social Work School of Health Sciences, Faculty of Biology, Medicine and Health The University of Manchester Manchester United Kingdom

**Keywords:** computerized physician order entry, CPOE, e-prescribing, system use, actual usage, systematic review

## Abstract

**Background:**

Computerized physician order entry (CPOE) systems in health care settings have many benefits for prescribing medication, such as improved quality of patient care and patient safety. However, to achieve their full potential, the factors influencing the usage of CPOE systems by physicians must be identified and understood.

**Objective:**

The aim of this study is to identify the factors influencing the usage of CPOE systems by physicians for medication prescribing in their clinical practice.

**Methods:**

We conducted a systematic search of the literature on this topic using four databases: PubMed, CINAHL, Ovid MEDLINE, and Embase. Searches were performed from September 2019 to December 2019. The retrieved papers were screened by examining the titles and abstracts of relevant studies; two reviewers screened the full text of potentially relevant papers for inclusion in the review. Qualitative, quantitative, and mixed methods studies with the aim of conducting assessments or investigations of factors influencing the use of CPOE for medication prescribing among physicians were included. The identified factors were grouped based on constructs from two models: the unified theory of acceptance and use of technology model and the Delone and McLean Information System Success Model. We used the Mixed Method Appraisal Tool to assess the quality of the included studies and narrative synthesis to report the results.

**Results:**

A total of 11 articles were included in the review, and 37 factors related to the usage of CPOE systems were identified as the factors influencing how physicians used CPOE for medication prescribing. These factors represented three main themes: individual, technological, and organizational.

**Conclusions:**

This study identified the common factors that influenced the usage of CPOE systems by physicians for medication prescribing regardless of the type of setting or the duration of the use of a system by participants. Our findings can be used to inform implementation and support the usage of the CPOE system by physicians.

## Introduction

### Background

Computerized physician order entry (CPOE) systems for medication prescribing allow health care professionals to enter accurate and complete medication orders electronically [1]. The CPOE system has clinical decision support (CDS) features that help reduce medication errors and increase safety, such as an alert system, to warn a physician of drug allergies and drug-drug interactions and a feature offering advice regarding medication dosages and frequencies [1]. CPOE for prescribing medication has been reported to be helpful to clinicians by providing them with easy access to patient data, a faster prescribing process [2], and guidelines to enhance compliance with best practices; it also reduces medical costs and improves organizational efficiency [3].

In addition to being beneficial for clinicians, CPOE for medication prescribing also has drawbacks that affect its usage by clinicians. Issues such as excessive alerting can lead physicians to ignore these safety warnings, which might be harmful for patients [4]. In addition, owing to the expense associated with continuous training required for such a system, physicians may lack adequate skills to use CPOE, which leads to underutilization [5].

The adoption and use of CPOE usually starts at the organizational level, where health organizations decide to implement such a system. Studies have shown that the adoption of CPOE for medication prescribing by health care organizations is associated with the high cost of installing a CPOE system. This may hinder many health care organizations from having a system within their practice. However, the benefits offered by the system in the long run can compensate for these costs [6].

For example, in 2013, a CPOE was implemented in 2 groups of 4 community hospitals in the United States at a cost of US $7,130,894 and US $19,293,379, respectively. After adopting the CPOE, the avoided financial cost of adverse drug events alone saves the hospital about US $7,937,651 and US $16,557,056 [7]. The organization makes the decision to implement the CPOE system; however, to achieve benefits and reach its full potential, CPOE depends on effective use by individual clinicians. There is a need to understand the factors influencing the usage of this system by physicians after it has been implemented. The aim of this review is to identify the factors that influence actual use of CPOE by physicians for medication prescribing.

The rationale for this systematic review was based on the results of previous studies, which suggested that the use of CPOE at the international level appears to be low [8-10]. The adoption of CPOE as a computerized ordering system for all types of medical orders (not only medication prescriptions) has international relevance [8,9]; however, evidence from studies conducted in several countries has shown a low rate of acceptance and adoption of these systems by health care providers [8,9]. For example, in some developing countries, despite the availability of several types of computerized health systems, such as electronic medical records, CDS systems, CPOE, and telemedicine, these systems are not properly used [9]. Although little has been reported in recent years about the proportion of CPOE users, in 2009 [8], the proportion of hospitals that implemented and adopted CPOE as an ordering system, including medication prescribing, in 7 western countries was reported. The study indicated that 15% of the hospitals in the United States, 2% in the United Kingdom, and 20% in the Netherlands had CPOE, with very few in Germany, France, and Australia. This shows a significantly low adoption rate [8], which was related to financial, organizational, and technological factors and attitudes of users [8].

In the United Kingdom, for example, vendors of CPOE systems for electronic prescribing have challenges related to implementation because of the factors related to policies [10]. In other countries with different health care systems and policies, the factors affecting the adoption and use of CPOE might vary.

### Objectives

The first rationale for conducting this study was to identify the factors influencing the underutilization of CPOE by physicians for medication prescribing and understand their reasons.

Second, we identified only 4 reviews with a main focus on CPOE as a medication-prescribing system [11-14]. The evidence from these reviews focused on the factors affecting health care providers during the implementation and adoption phases, rather than their actual use of CPOE postimplementation. The implementation phase refers to the time between deciding to introduce a new system and the activities involved in this decision by the hospital, up to the point the system is ready to be used [11]. In this study, we aim to identify the factors affecting the actual use of CPOE.

The actual usage of a system follows the implementation process [15]: actual usage is defined as a behavior that can be measured through indicators, such as an individual’s frequency or duration of usage [16]. The term system usage consists of 3 fundamental components: the subject using the system (user), the system itself, and the task to be accomplished through the system [17]. Although one of the reviews [14] focused on medication-related CDS after it was fully implemented, it included evidence only from qualitative studies, and there was no indication that the actual usage, as defined here, was the main focus of that review.

Two of the reviews [11,12] identified factors influencing different types of health care providers as users (eg, physicians, nurses, pharmacists), whereas the other 2 reviews [13,14] identified their targeted users. This study focused entirely on physicians as users and the factors that were likely to affect their usage, as professionals from different disciplines might be influenced by different factors in their decisions to use CPOE for prescribing medication. Hence, the second rationale for conducting this study was to fill the gap in the evidence found in prior reviews.

Third, most of the studies included in these reviews were conducted in industrialized western countries (the United States, the United Kingdom, Sweden, the Netherlands, Australia, and Canada); only 1 study was conducted in a developing country. There is a huge gap in the literature on the factors affecting the usage of CPOE for prescribing medication among developing countries [9]. This study was part of a research project conducted in Saudi Arabia (a developing country) to investigate the factors that influence the actual usage of CPOE by physicians for medication prescribing.

In summary, the aforementioned gap in the literature regarding the factors influencing the actual use of CPOE for medication prescribing by physicians is the reason for carrying out this systematic review. In this study, we used the unified theory of acceptance and use of technology (UTAUT) model [18] and the Delone and McLean Information System Success Model [19] as frameworks to classify the evidence on the actual use of CPOE by physicians for medication prescribing. To the best of our knowledge, there is no published analysis of the factors affecting the actual use of CPOE in particular by physicians for medication prescribing using this theoretical approach.

## Methods

### Search Strategy

This study was based on the PRISMA (Preferred Reporting Items for Systematic Reviews and Meta-analyses) guidelines [20]. The following databases were searched from September 2019 to December 2019: PubMed, Embase, Ovid MEDLINE, and CINAHL. The search was performed without any restrictions on dates; however, it was limited to English language papers. Reference lists in the identified reviews and included studies were checked to retrieve relevant papers. We combined medical subject headings (MeSH terms) related to CPOE retrieved from PubMed and keywords from the relevant research literature (Textbox 1).

**Medical subject headings (MeSH) terms and keywords used in the searches of PubMed, Embase, Ovid MEDLINE, and CINAHL. The final search strategy (A10, B8, and C3) was applied to all 4 databases.**

Group A: type of system

1. Medication alert systems
2. Computerized provider order entry
3. Computerized physician order entry
4. CPOE
5. Electronic prescription
6. Prescription decision support system
7. Computerized prescriber order entry
8. Pharmaceutical decision-support systems
9. Pharmacy information system
10. 1 or 2 or 3 or 4 or 5 or 6 or 7 or 8 or 9

Group B: usage

1. Use
2. Actual usage
3. System use
4. Utilization
5. Acceptance
6. Adoption
7. Usage
8. 1 or 2 or 3 or 4 or 5 or 6 or 7

Group C: factors

1. Factors
2. Determinants
3. 1 or 2

A draft of the search strategies used in three of the databases is presented in Multimedia Appendix 1.

### Eligibility Criteria

The included studies were peer-reviewed research reports written in English, with the stated aim of exploring, investigating, or assessing factors that influence the use of medication-related CPOE systems as our target intervention. The population of interest was physicians, with the included studies reporting the results of physicians only or papers in which physicians’ responses were reported separately. The included studies also had to be conducted in clinical settings, that is, inpatient and outpatient departments of hospitals, health care centers, primary care centers, and polyclinics. Quantitative, qualitative, and mixed methods designs were considered eligible for inclusion. Studies were excluded if the CPOE system had not been implemented at the time of this study or if the study assessed the influence of factors on intentions to use the CPOE system rather than on its actual use. Papers with a population of nurses, pharmacists, information technology (IT) personnel, managers, or patients and those with interventions that were not strictly CPOE, as defined earlier, were excluded from the review. Studies that were conducted in nonclinical settings (eg, retail pharmacies, community pharmacies, nursing homes) were excluded from this review.

### Selection Process

**Figure 1:**
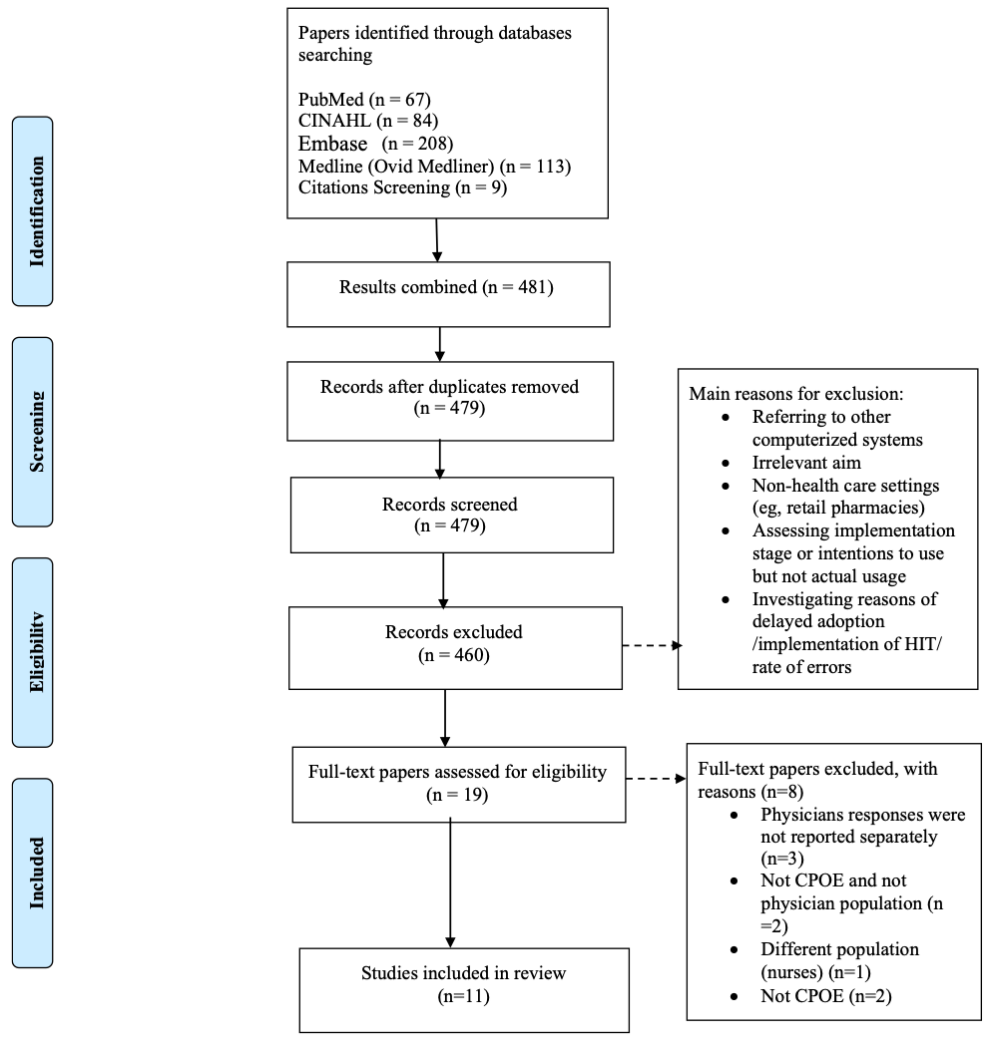
Flow diagram of the selection process for the included papers. CPOE: computerized physician order entry; HIT: health information technology.

The primary researcher (AM) independently screened the titles and abstracts of all papers retrieved from the search using the inclusion criteria. The full-text articles of all potentially relevant studies were assessed independently by all 3 authors for eligibility. A calibration exercise was conducted to cross-check the results obtained by the authors. All disagreements were resolved through discussion. The details of the exclusion criteria are shown in Figure 1.

### Data Collection Process and Data Items

The primary researcher performed the data extraction. The data included names of the authors, publication year, country, objective, study design, data collection method, type of intervention, setting, population and sample, factors associated with CPOE use, how actual use was assessed, and the duration of the system’s use before the data were collected.

### Risk of Bias of the Included Studies (Quality Assessment)

The Mixed Methods Appraisal Tool (MMAT) was used to assess the quality of the included studies [21]. The MMAT is a comprehensive tool designed to evaluate reviews, including quantitative, qualitative, and mixed methods studies [21]. All the 3 authors independently appraised the included studies. The primary researcher (AM) reviewed all of the studies, and each of the other 2 researchers (JA and DD) reviewed half of the studies. Any disagreements were resolved through discussion. MMAT does not recommend assigning a single score based on the assessment [21]. However, in this review, we used a specific metric derived from a previous study [22]. To rate the quality of each of the studies to justify the reasons for the final inclusions and exclusions. Studies were classified as high, medium, or low quality, depending on the number of criteria that were met. A study was considered high quality if all 5 MMAT criteria were met, medium if 3 or 4 criteria were met, and low when a study met 1 or 2 criteria [22].

### Data Synthesis

Narrative synthesis was used to summarize the evidence from the included studies. Narrative synthesis is appropriate when a review includes both qualitative and quantitative findings [23].

## Results

### Study Selection

The electronic database search retrieved 67 records from PubMed, 84 from CINAHL, 208 from Embase, 113 from Ovid MEDLINE, and 9 from the reference lists of the included studies. After duplicates were removed, the titles and abstracts of the remaining 479 studies were assessed for eligibility. Of these, 460 studies were excluded because they were ineligible and 19 articles were selected for in-depth analyses. A total of 11 studies were included in the final review. The study selection process and reasons for exclusion are shown in Figure 1.

### Characteristics of the Included Studies

Multimedia Appendix 2 [24-34] summarizes the characteristics of the included studies. The 11 studies included in the review were from different regions of the world: 4 are from the United States [24-27], 3 are from Sweden [28-30], 1 is from the Netherlands [31], 1 from Saudi Arabia [32], 1 from Australia [33], and 1 from Singapore [34]. Of the total number of studies, 4 used qualitative methods (interviews) [24,25,29,33], 6 used quantitative methods (surveys or questionnaires) [26-28,30,32,34], and 1 used a mixed methods approach [31]. Among the 11 included studies, the factors associated with the use of CPOE for medication prescribing were mainly related to technical, organizational, or individual characteristics. All the included studies were conducted in either a hospital or a primary care center. Of the total number of studies, 7 were conducted in a hospital setting [24-27,29,32,33], 2 in a hospital and a primary care center [28,30], 1 in a primary care center [31], and another in a group of polyclinics [34].

### Quality of the Included Studies

Multimedia Appendix 3 [24-34] summarizes the results of the quality assessment of the included studies. Of the total number of studies, 3 (all qualitative) were rated as *high* quality because they met all 5 MMAT criteria [24,25,29]. Of the total number of studies, 5 (all quantitative) were rated as *medium* quality, as they met 3 or 4 of the MMAT criteria [26,28,30,32,34] and 3 studies were evaluated as having low quality because they met either 1 or none of the MMAT criteria. Of these, 1 was a quantitative study [27], 1 study used a mixed methods design [31], and 1 was a qualitative study [33]. We chose not to exclude these studies from the final synthesis based on their quality because of the exploratory nature of the review.

### Synthesis of the Results

The factors that influenced physicians’ usage of CPOE for medication prescribing are presented in Table 1. On the basis of the perceived commonality among the reported factors, we organized them according to the definitions of the constructs from the UTAUT [18] and the Delone and McLean Information System Success Model [19].

**Table 1:** Factors influencing the frequency of use of the computerized physician order entry system by physicians.

Theme, construct, and factor			Studies, n	Study
**Individual factors**				
	**Performance expectancy: perception that using CPOE^a^ will improve the physician’s job performance [18]**			
		Perceived usefulness	1	[29]
		Relative advantage	1	[30]
		Effect on quality of care and/or patient outcomes	3	[25,26,32]
		Effects on productivity	2	[25,34]
		Effects on safety	1	[24]
		Performance outcomes	1	[25]
	**Effort expectancy: belief that the CPOE is easy to use [18]**			
		Ease of use	3	[28,29,32]
		User-friendliness	1	[31]
		Difficult to use	2	[24,25]
		Complexity	1	[30]
	**Social influence: perceived importance of others’ (eg, leaders, colleagues) opinions that the physician should or should not use the system [18]**			
		External normative beliefs	1	[25]
**Organizational factors**				
	**Facilitating conditions: available resources, facilities, and infrastructure that facilitate using CPOE [18]**			
		Training	4	[24,25,33,34]
		Availability of technical support	4	[25,27,31,32]
		Compatibility	1	[30]
		Computer skills	1	[34]
		Time constraints	3	[24,25,27]
		Availability of hardware	2	[25,27]
		Lack of awareness of the availability of certain features	1	[33]
		Management support	1	[25]
		User involvement	1	[25]
**Technological factors**				
	**Information quality: relevance, accuracy, comprehensiveness, understandability, prevalence, timeliness, and usability of the outputs or content [19]**			
		Usefulness of error messages	1	[32]
		Clarity and brevity of the reminders	1	[31]
		Confidentiality, privacy, and security of patients’ records	1	[25]
	**System quality: reliability, functionality, flexibility, ease of use, integration, and response time of the system [19]**			
		Clarity	2	[28,32]
		Layout	1	[31]
		Technical problems causing delays during prescribing	1	[31]
		System’s speed	3	[31,32,34]
		Software barriers	1	[25]
		Reliability	1	[32]
		Customization to individual departments	2	[25,33]
		Functionality of the tools in the system	1	[34]
		Locating items on the system	1	[32]
		Retrieval of radiology data	1	[32]
		Usability	1	[24]
		System’s efficiency	2	[24,26]
		Availability of reference materials	1	[32]
		Alert fatigue	2	[24,33]

^a^CPOE: computerized physician order entry.

UTAUT is a theoretical model that can explain about 70% of the variance in a user’s behavior in relation to technology acceptance and use [18]. It consists of 4 main constructs: performance expectancy, effort expectancy, social influence, and facilitating conditions [18]. Performance expectancy refers to physicians’ perceptions that using CPOE will improve their job performance [18]. Effort expectancy refers to physicians’ beliefs that using CPOE is effortless and easy [18]. Social influence pertains to physicians’ perceptions of the importance of others’ (eg, leaders’ and colleagues’) opinions about whether physicians should or should not use the system [18]. Facilitating conditions refers to the existence of resources, facilities, and infrastructure that are helpful to physicians when using CPOE [18].

The Delone and McLean Information System Success Model is used to assess and understand the success of any information system and its impact on the individual and the organization [19]. It consists of 6 components: system quality, information quality, use, user satisfaction, individual impact, and organizational impact [19]. However, we assessed only system quality and information quality. Information quality refers to the system’s outputs or content in terms of relevance, accuracy, comprehensiveness, understandability, prevalence, timeliness, and usability [19]. System quality refers to the quality of the system, in particular, the system’s reliability, functionality, flexibility, ease of use, integration, and response time [19]. We assessed these 2 constructs because the identified factors that are mainly related to the technological aspects of the CPOE system are also related to the quality of the information and the system. The other 4 constructs were addressed in the UTAUT model.

The results of the included studies were synthesized under 3 themes: individual, organizational, and technological factors. Individual factors are related to the constructs of performance expectancy, effort expectancy, and social influence. Organizational factors are related to the construct of facilitating conditions, and technological factors are related to the constructs of information quality and system quality (Table 1).

### Individual Factors

Individual factors refer to issues related to physicians’ perceptions of the possible effects of using CPOE for medication prescribing [35]. A total of 11 factors related to physicians’ perceptions were identified. The most cited factors were the effect on the quality of patient care [25,26,32] and ease of use [28,29,32]. Physicians perceived that using CPOE enhanced patient care. In one study [26], the features of the CPOE system were associated with better quality of patient care by providing easy and direct access to patient records and reminders and alerts for physicians, which led to a reduction in duplicate tests and expediting the ordering process. Ease of use refers to physicians’ belief that using the system is easy and effortless [18,28,29]. In another study [32], physicians agreed that their satisfaction with the system was greater because it was easy to use, which led to their usage of the system. Three studies reported limited use of CPOE by physicians because they found it difficult to use and complex in terms of navigating, accessing, and finding information [24,29,30].

### Organizational Factors

Organizational factors include resources (eg, materials, humans, circumstances) provided by the organization that facilitate usage of the CPOE system by physicians [12]. In total, 8 studies identified 9 organizational factors that affected the use of CPOE. Training [24,25,33,34], availability of technical support (such as a help desk) [25,27,31,32], and time constraints [24,25,27] were the most cited factors. Training issues reported by physicians included either the need for retraining because of new features [24] or lack of training [33]. The availability of technical support means the physicians need to have IT staff accessible to help them in case of any technical issues while using the CPOE system [25,27,32] or the extent of the physician’s awareness that there is a designated help desk to assist them [31].

The timing of the reporting of these factors in the included studies suggests that the factors related to the organization were critical for the usage of the CPOE system by physicians, regardless of whether the physicians recently began using the system or have been using it for a longer time. For example, studies that reported training [24,25,33,34] were conducted at different time points after the implementation of CPOE. One study conducted its assessment after 2 years of CPOE usage [24], while 3 other studies investigated the factors affecting usage after only months of use [25,33,34]. Technical support availability was reported in studies after weeks [25,31,32] and after 1 year of usage [27].

Time constraints were the second most cited factor influencing physicians’ CPOE usage [24,25,27]. The complexity of CPOE [24], its slowness [25], and physicians’ unfamiliarity with its features [27] were reasons why it was so time-consuming for physicians to use it.

### Technological Factors

Technological factors included the technical and design aspects of CPOE in terms of the system’s quality; information quality; and its reliability, functionality, flexibility, ease of use, integration, and response time [19]. Evidence from 8 of the included studies [24-26,28,31-34] indicated that the factors related to CPOE were the most relevant for affecting its use by physicians. A total of 17 factors were reported (Table 1). The system’s efficiency was the most cited factor [31,32,34], specifically the quick prescribing process [31], fast data retrieval, response time [32], and the system’s speed, in terms of entering patient data [34]. Furthermore, studies that reported the system’s speed as an influential factor in its use by physicians were conducted shortly after the implementation phase, that is, halfway through the intervention year (about 6 months later), shortly after implementation (not clear), and 3 months after implementation. This finding suggests that because the system was newly implemented, the processing speed was significant for physicians’ performance of tasks.

The findings indicate that ease of use, the effect of using CPOE on quality of care, training, availability of technical support, time, and the system’s speed were the factors with the strongest influence on the use of CPOE for medication prescribing among all the studies.

## Discussion

### Principal Findings and Comparisons With Other Works

CPOE for medication prescribing can serve physicians as a tool to enhance patient quality of care. However, this has not led to a rapid uptake of the system by health organizations and clinicians to use it [6,14]. A key factor in the slow adoption of CPOE by health care organizations is attributed to the costs associated with installing the system and the costs of sustaining it [6]. The first CPOE was installed in the United States in 1971 [36]. Although that was long ago, the adoption rate in health organizations is still rare to moderate, with a percentage of 15.7% [13]. This low adoption rate has been reported in other countries [8,9].

Despite many years of implementation of CPOE for medication prescription, development, and research, the issue of low adoption postimplementation remains. This study focuses on the usage of the user—the physician—after the system has been implemented. We identified factors that were related to the users (physicians), organization, and technological aspects of CPOE that influence the actual use of CPOE by physicians for medication prescribing, rather than intention to use a CPOE system.

The findings of this study are consistent with those of Van Dort et al [14] and Gagnon et al [12]. Nevertheless, these reviews identified other factors that were not found in this study. Resistance to use was reported in both reviews [12,14], as a factor that negatively affected the usage of the system by physicians for medication prescribing. CDS systems embedded in the CPOE system for medication prescribing were examined in Van Dort et al [14]. As CDS systems are known to offer suggestions and recommendations, user resistance was present as the physicians reported concerns that the information presented might not be reliable [14].

In addition to resistance to using CPOE, Gagnon et al [12] described how the system could negatively affect the patient-clinician relationship and identified financial issues as another influential factor, neither of which was detected in this study. This inconsistency might be because of the focus of this study on the actual use of CPOE after the system had been installed and used and resistance is no longer an issue.

This study showed that technological factors related to the system were the most frequently reported factors that influenced how a physician used the CPOE system for medication prescribing. This finding is consistent with the results reported by Gagnon et al [12]. As their findings suggest, technical and design concerns were the most frequently identified factors limiting the system’s use [12].

One of the principal findings of this study is that among the 3 main themes, 5 factors were cited most frequently (any factor cited 3 or more times was considered frequently cited), indicating that it was significant in the physicians’ decisions about using the CPOE system. Quality of care, ease of use, training, availability of technical support, time constraints, and system speed were key factors in the use of CPOE by physicians. A similar pattern of results has been reported in an extensive body of literature [12,14,37,38]. One unexpected finding was that the effect of alert fatigue, as a factor in the use of CPOE, was identified in only 2 studies [24,33]. Alert fatigue is the receipt of a massive amount of reminders or warnings that cost time and effort and is eventually ignored [39].

This finding contradicts the observation that alert fatigue has previously been found to be associated with the usage of CPOE for medication prescribing. In their review, Gagnon et al [12] showed that alert fatigue was associated with the use of an electronic prescription system in 5 studies. In addition, Van Dort et al [14] showed that too many irrelevant alerts were related to the uptake of medication-related CDS systems in 10 studies.

In these 2 studies [24,33], alert fatigue affected physicians’ use. In the first study [24], physicians’ perception of the alerts was that after transitioning to a more advanced new system, the alerts were more sensitive than those of the older system. In the second study [33], the ratings of the alerts were higher when the study’s setting was an intensive care unit (ICU), compared with their ratings by other departments in the hospital.

All factors identified in this study are similar to those of other reviews related to the implementation [12], adoption [37], or acceptance [38] of CPOE.

However, a factor not discussed in previous CPOE for e-prescription studies and detected in this study was customization of the CPOE system’s features for medication prescribing to each department. Customize refers to tailoring the features of a CPOE system to the preferences and needs of a specific department. For example, ICU physicians reported that some alerts were irrelevant to ICU patients and more suitable for other departments in the hospital [33]. This finding is in line with that reported in the review by Li et al [40], who suggested the importance of customization of the system’s features according to different specialties and emphasized its significance for the provider’s workflow.

We have used constructs from the UTAUT [18] and Delone and McLean Information System Success Models [19] to organize the identified factors to provide a better understanding of what each factor means to the user and how it may influence physicians’ attitudes toward the actual use of the CPOE for medication prescribing. The UTAUT model is a combination of 8 technology acceptance models, which covers almost all the factors identified in the literature [18]. All the factors reported in the included literature in this study were aligned with the constructs of the UTAUT and Delone and McLean Information System Success Models. The examination of factors using these 2 models provides a useful framework for this systematic review.

Two of the constructs (system quality and information quality) from the Delone and McLean Information System Success Model were found to be highly relevant, as the most frequently reported factors were the technological ones [19]. These factors were mainly related to the quality of the system or information. Both models have been extensively used in research related to health care technology assessment [41,42].

### Limitations and Strengths

The limitations of this study should be acknowledged. First, we searched only 4 databases. Although these databases are the most relevant for health care publications, there is a possibility that relevant studies could have been missed. Second, the first step of the database search—checking every single title and abstract—was performed by a single author. However, we believe that this does not affect the quality of this paper as the results of the selection and screening were revised in regular meetings with the other reviewers who are experts in the field and no issues were raised by them during the review process. In addition, all the assessment steps for article eligibility were conducted by all 3 authors in parallel. We systematically discussed any disputes between all the reviewers to ensure consistency.

Third, we acknowledge the fact that our search resulted in only 11 articles that could be viewed as a small sample for a system that has been in use for a number of years. However, this study focused on the medication ordering aspect of the CPOE and did not evaluate the CPOE as a whole system. In addition, we also focused on physicians as our target population and studies that indicated that the system is being actually used and not the intention to use (installation phase or implementation phase). The strength of this study lies in the presentation of 4 elements that are absent from previous attempts to synthesize primary research on this topic: (1) it evaluated research that used major study designs (quantitative, qualitative, and mixed methods); (2) it drew on the perspectives of physicians only; and (3) it included research on the period of actual usage of CPOE for e-prescribing in particular (while the physicians were using the system) and not the intention to use. (4) Factors that are unique to the physician’s actual usage were explained using a framework that consists of a combination of 2 theoretical approaches. To the best of our knowledge, no previous systematic reviews have explored specific factors influencing physicians’ actual usage of CPOE or e-prescriptions according to the presented framework.

### Conclusions

This study suggests that an individual’s perceptions, technical factors, and organizational factors are all significant influences on the usage of CPOE by physicians for medication prescribing. Although most of the identified factors are similar to those reported in previous reviews related to CPOE, the results of our work have allowed us to identify an additional factor that was not discussed in earlier reviews, namely, the preference of physicians to customize the CPOE system to the needs of the medical department. Finally, as much as there are issues at the organizational level during the implementation process, it is important to focus on the individual physicians after the implementation is completed. The outcomes of this study provide a source of knowledge for health care decision makers, managers, and staff and a clear understanding of the factors influencing the usage of CPOE by physicians for medication prescribing, which can inform future system designs and implementation.

## Appendix

Multimedia Appendix 1 Results of the search strategies used in the PubMed, EMBASE, and CINAHL databases.

Multimedia Appendix 2 Characteristics of the included studies.

Multimedia Appendix 3 Quality assessment of the included studies using the Mixed Methods Appraisal Tool (2018).

## Abbreviations

CDS: clinical decision support
CPOE: computerized physician order entry
ICU: intensive care unit
IT: information technology
MMAT: Mixed Methods Appraisal Tool
UTAUT: unified theory of acceptance and use of technology
