# Supplementary material for: Physicians’ Use of the Computerized Physician Order Entry System for Medication Prescribing: Systematic Review
Source: JMIR Med Inform. 2021 Mar 4;9(3):e22923. doi: 10.2196/22923 (PMC7974763; doi:10.2196/22923)
Supplement: Multimedia Appendix 1 [file medinform_v9i3e22923_app1.docx]

**Multimedia Appendix 1.** Results of the search strategies used in PubMed, EMBASE, and CINAHL.

**PubMed**

| Search | Results |
| --- | --- |
| 1. (Medication Alert Systems[Title/Abstract] OR Computerized Provider Order Entry[Title/Abstract] OR Computerized Physician Order Entry[Title/Abstract] OR CPOE[Title/Abstract] OR Electronic Prescription[Title/Abstract] OR Prescription decision support system[Title/Abstract] OR computerized prescriber order entry[Title/Abstract] OR pharmaceutical decision support systems [Title/Abstract] OR Pharmacy information system[Title/Abstract]) Sort by: Best Match | 2443 |
| 1. (actual use[Title/Abstract] OR Use[Title/Abstract] OR System use[Title/Abstract] OR utilization[Title/Abstract] OR Acceptance[Title/Abstract] OR Adoption[Title/Abstract]) Sort by: Best Match | 283962 |
| 1. (Factors[Title/Abstract] OR determinants[Title/Abstract]) Sort by: Best Match | [2054241](https://www.ncbi.nlm.nih.gov/pubmed/?cmd=HistorySearch&querykey=7) |
| 1. ((((Medication Alert Systems[Title/Abstract] OR Computerized Provider Order Entry[Title/Abstract] OR Computerized Physician Order Entry[Title/Abstract] OR CPOE[Title/Abstract] OR Electronic Prescription[Title/Abstract] OR Prescription decision support system[Title/Abstract] OR computerized prescriber order entry[Title/Abstract] OR pharmaceutical decision support systems [Title/Abstract] OR Pharmacy information system[Title/Abstract]))) AND ((actual use[Title/Abstract] OR Use[Title/Abstract] OR System use[Title/Abstract] OR utilization[Title/Abstract] OR Acceptance[Title/Abstract] OR Adoption[Title/Abstract]))) AND ((Factors[Title/Abstract] OR determinants[Title/Abstract])) Sort by: Best Match | 67 |
|  |  |

**EMBASE**

| Search | Results |
| --- | --- |
| 1. (actual usage or Use^a^ or System use^b^ or utilization or Acceptance or Adoption).ab. | 8554140 |
| 1. (factors or determinants).ab. | 2547366 |
| 1. (Medication Alert Systems or Computerized Provider Order Entry or Computerized Physician Order Entry or CPOE or Electronic Prescription or Prescription decision support system or computerized prescriber order entry or pharmaceutical decision support systems or Pharmacy information system).ab. | 2648 |
| 1. 1 and 2 and 3 | 217 |
| 1. limit 4 to English language | 208 |

**CINAHL**

| Search | Results |
| --- | --- |
| 1. AB Medication Alert Systems OR Computerized Provider Order Entry OR Computerized Physician Order Entry OR CPOE OR Electronic Prescription OR Prescription decision support system OR computerized prescriber order entry OR pharmaceutical decision support systems OR Pharmacy information system | 1137 |
| 1. AB actual usage OR Use OR System use OR utilization OR Acceptance OR Adoption | 601932 |
| 1. AB factors OR determinants | 515609 |
| 1. S1 AND S2 AND S3 | 84 |

^a^actual usage or Use: The CPOE system in the study is already installed and physicians are practically using it . Not intended to be used or still not installed.

^b^System use: the utilization of the CPOE system
