# Supplementary material for: Physicians’ Use of the Computerized Physician Order Entry System for Medication Prescribing: Systematic Review
Source: JMIR Med Inform. 2021 Mar 4;9(3):e22923. doi: 10.2196/22923 (PMC7974763; doi:10.2196/22923)
Supplement: Multimedia Appendix 2 [file medinform_v9i3e22923_app2.docx]

**Multimedia Appendix 2.** Characteristics of the included studies.

| **Author/Year** | **Country** | **Objective** | **Study Design/Data Collection Methods** | **Intervention** | **Setting** | **Population/Sample Size** | **Factors Associated with Usage** | **Actual Usage Reporting** | **Duration of System Usage at the Time of the Study** |
| --- | --- | --- | --- | --- | --- | --- | --- | --- | --- |
| Abramsom et al, 2016 ^[24]^ | US | To evaluate how physicians’ perceptions and experiences with prescribing evolved after prolonged system use | Qualitative/Semi-structured interviews | Electronic prescribing system | Hospital-based adult internal medicine outpatient clinic | 13 Physicians (only 11 were interviewed). | - Efficiency and usability - Effects on safety - Training - Alert fatigue - Shortcut features would either be too difficult to customize and time consuming | Not applicable^a^ | For 2 years |
| Hellström et al, 2009 ^[28]^ | Sweden | To assess experienced e-prescribers' attitudes toward e-prescribing for suggesting improvement | Quantitative/Survey | EHR^b^ systems with integrated electronic prescribing modules | Primary  care centers  & hospitals  (primary care, internal medicine, orthopedics, and general surgery) | 431 Physicians  (180/431 – 42% ) | - Ease of use - Clarity of information display | Number of E-Prescriptions per day (self-reported) | 15% of the respondents had used an electronic system for two months to one year, and 85% for more than one year |
| Holden, 2010  ^[25]^ | US | To describe physicians' beliefs about the use of EMR^c^ and CPOE^d^ for inpatient and outpatient care, to identify what factors shaping information technology usage | Qualitative/Semi-structured interviews | EMR^J^ and CPOE | 2 Hospitals (inpatient/outpatients) | 20 Physicians | - Performance outcomes - Productivity and efficiency outcomes - Behavioral beliefs - Financial, organizational factors - Normative beliefs - Moral normative beliefs - Hardware and software barriers - Environmental barriers - Insufficient time to use the system or to learn to use it - Availability of training and technical support facilitated use | Not applicable | H^e^1: provided data on the first few weeks of using CPOE  H2: about 7 months |
| Martens et al., 2008 ^[31]^ | The Netherlands | To evaluate the feasibility and acceptability of a CRS^f^ to improve prescribing behavior and to investigate the strengths and weaknesses of a reminder system | Mixed methods  Quantitative/Questionnaire  Qualitative/Semi-structured interviews | CRS | Primary care practice | 53 Physicians  2 Project leaders  1 Technical consultant | - Stability and speed of the CRS - Instructiveness and shortness of the reminders, - User-friendliness - Layout - Support from the help desk. - Technical problems that caused delay during prescribing | Mean number of reminders per GP^f^ per month per 1000 enlisted patients through (system logs) | Halfway through the intervention year |
| Omar, 2016 ^[29]^ | Sweden | To study pediatrician’s attitude towards EPDSS^g^ and to investigate factors affecting user acceptance using a technology acceptance model | Qualitative/Semi-structured interviews | Electronic prescribing decision support system | Hospital pediatric department | 7 Physicians | - Perceived usefulness - Perceived ease of use | Not applicable | 2 to 4 years |
| Rahimi et al , 2009 ^[30]^ | Sweden | To observe factors associated with the adoption of a CPOE system for inter-organizational and intra-organizational care using the diffusion of innovation theory | Quantitative/Survey | CPOE | Primary healthcare centers and hospitals using the system in Östergötland County | 741 Physicians (176/741 physicians - 23.8%)  200 Nurses (134/200 nurses - 67.0%) | - Relative advantage - Compatibility - Complexity | Number of entered orders in the CPOE system in a normal day (self-reported) | After 1 year |
| Saddik & Al- Fridan, 2012 ^[32]^ | Saudi Arabia | To measure the satisfaction of physicians toward CPOE and explore the factors associated with satisfaction | Quantitative/Questionnaire | CPOE  . | Hospital (all units) | 101 Physicians (81/101 – 80%) | - Ratings of impact on patient care and quality, speed, clarity, and reliability - Characteristics related to locating items on the clarity of correcting mistakes - Ease of use. - Availability of technical support, reference materials, and the usefulness of error messages - Locating items in the system - Retrieval of radiology data | Not applicable | Shortly after implementation (Not clear how shortly) |
| Santucci et al, 2016 ^[33]^ | Australia | To determine whether physicians in ICUs use and perceive hospital-wide CDS^h^ useful for integration with an electronic prescribing system | Qualitative/Observa-tion and interviews | Electronic prescribing system | Hospital - 12-bed general/neuro-logical intensive care unit | 20 Physicians | - Customization of pre-written orders for the ICU context - Alerts/alarm fatigue - Unawareness that the reference viewer tool was available - Insufficient training | Through shadowing 20 doctors | 4 Months  (The study reported that at the time of the study, June–September 2014, the electronic prescribing system was in use in all wards of the hospital, including the ICU^H^ |
| Schectman et al , 2005 ^[26]^ | US | To understand whether physicians’ computer experience and attitudes or other barriers were related with the actual adoption of an expert prescription system | Quantitative/System’s logs and survey | Prescription expert system | Academic internal medicine residency training clinic | 94 Physicians ( 84/94 - 89% ) | - System efficiency - Effect on quality | -System utilization rate : the number of electronic prescriptions written by each physician during the study period (system logs)  -self reported survey. | 6 months post-implementation |
| Shriner & Webber, 2014 ^[27]^ | US | To explore residents’ perceptions and attitudes toward implementation of CPOE CDS prior to implementation and at 6 months and 12 months post-implementation | Quantitative pre-implementation and post implementation survey | CPOE | Pediatric hospital | 146 Physicians  43.8% (n=64) of residents responded to the pre-CPOE survey  37.6% (n=55) responded to the 6-month post-CPOE survey  43.2% (n=63) responded to the 12-month survey. | - Time constrains (at both 6- and 12-months assessment). - The degree of EMR implementation; support staff availability - Hardware availability | Not applicable | After 6 months then after 12 months |
| Tan et al , 2009 ^[34]^ | Singapore | To assess users’ satisfaction and factors associated with satisfaction toward the electronic prescription system | Quantitative/Questionnaire | CPOE | Polyclinics | 118 Doctors  61 Pharmacy staff | - Computer skills - Functionality (detection of prescribing errors and the ability to receive alerts for drug-interactions and drug-allergies) - Processing (entering orders) and system speed - Training and ongoing support - Effect on productivity | Not applicable | After 3 months |

^a^Not applicable: not a part of this study

^b^EHR: Electronic health record

^c^CPOE: Computerized physicians’ order entry

^d^H: Hospital

^e^CRS: Computer reminder system

^f^GP: General practitioner

^g^EPDSS: Electronic prescribing decisions support system

^h^CDS: Computerized decision support; the decision support system is part of the computer reminder system, which is embedded in the existing GP information system that includes the features of the decision support system for drug prescribing.

^i^ICU: Intensive care unit

^j^EMR: Electronic medical record
