# Supplementary material for: Physicians’ Use of the Computerized Physician Order Entry System for Medication Prescribing: Systematic Review
Source: JMIR Med Inform. 2021 Mar 4;9(3):e22923. doi: 10.2196/22923 (PMC7974763; doi:10.2196/22923)
Supplement: Multimedia Appendix 3 [file medinform_v9i3e22923_app3.docx]

**Multimedia Appendix 3.** Quality assessment of the included studies using the MMAT (2018)^a^.

|  | **1.^b^ Qualitative** | | | | | **4. Quantitative Descriptive** | | | | | **5. Mixed Methods** | | | | | **Quality of the Study** |
| --- | --- | --- | --- | --- | --- | --- | --- | --- | --- | --- | --- | --- | --- | --- | --- | --- |
| **References in Alphabetical Order** | **1.1^c^** | **1.2** | **1.3** | **1.4** | **1.5** | **4.1** | **4.2** | **4.3** | **4.4** | **4.5** | **5.1** | **5.2** | **5.3** | **5.4** | **5.5** |  |
| Abramsom et al, 2016 ^[24]^ | Yes | Yes | Yes | Yes | Yes |  |  |  |  |  |  |  |  |  |  | High |
| Hellström et al, 2009 ^[28]^ |  |  |  |  |  | Yes | Yes | No | No | Yes |  |  |  |  |  | Medium |
| Holden 2010 ^[25]^ | Yes | Yes | Yes | Yes | Yes |  |  |  |  |  |  |  |  |  |  | High |
| Martens et al., 2008 ^[31]^ | Yes | Can’t Tell | No | No | No | Yes | No | Yes | Yes | Yes | No | No | No | No | No | Low |
| Omar, 2016 ^[29]^ d | Yes | Yes | Yes | Yes | Yes |  |  |  |  |  |  |  |  |  |  | High |
| Rahimi et al , 2009 ^[30]^ |  |  |  |  |  | Yes | Yes | Yes | No | Yes |  |  |  |  |  | Medium |
| Saddik & Al- Fridan , 2012 ^[32]^ |  |  |  |  |  | Yes | Yes | Can’t Tell | Yes | Yes |  |  |  |  |  | Medium |
| Santucci et al,2016 ^[33]^ | Can’t Tell | Can’t Tell | Yes | No | Can’t Tell |  |  |  |  |  |  |  |  |  |  | Low |
| Schectman et al , 2005 ^[26]^ |  |  |  |  |  | Yes | Yes | Can’t Tell | Yes | Yes |  |  |  |  |  | Medium |
| Shriner & Webber, 2014 ^[27]^ |  |  |  |  |  | No | No | No | No | Yes |  |  |  |  |  | Low |
| Tan et al , 2009 ^[34]^ |  |  |  |  |  | Yes | Yes | Yes | No | Yes |  |  |  |  |  | Medium |

^a^MMAT: Mixed Methods Appraisal Tool

^b^1., 4., 5.: Sections of the MMAT used to evaluate the qualitative, quantitative and mixed–methods studies, respectively

^c^1.1-5.5: Items in each of the MMAT sections used to evaluate the qualitative, quantitative and mixed-methods studies

^d^Due to the high relevance of this paper, we used the primary source, which was a master’s degree research paper, and not the article that was identified through the systematic search, as we were reporting it in this review. The primary researcher Googled the title and retrieved the primary source:<https://pdfs.semanticscholar.org/d7c6/40411a6bc8f7f2ef3d504bee91b2208893e8.pdf>
